# Small molecule p300/catenin antagonist enhances hematopoietic recovery after radiation

**DOI:** 10.1371/journal.pone.0177245

**Published:** 2017-05-09

**Authors:** Yi Zhao, Kaijin Wu, Cu Nguyen, Goar Smbatyan, Elisabeth Melendez, Yusuke Higuchi, Yibu Chen, Michael Kahn

**Affiliations:** 1 Department of Medicine, Keck School of Medicine of University of Southern California, Los Angeles, California, United States of America; 2 Center for Molecular Pathways and Drug Discovery, Keck School of Medicine, University of Southern California, Los Angeles, California, United States of America; 3 Norris Comprehensive Cancer Center, University of Southern California, Los Angeles, California, United States of America; 4 Department of Biochemistry and Molecular Biology, Norris Comprehensive Cancer Research Center, Keck School of Medicine of University of Southern California, Los Angeles, California, United States of America; 5 Department of Organic Fine Chemicals, The Institute of Scientific and Industrial Research, Osaka University, Ibaraki, Osaka, Japan; 6 Bioinformatics Service Program, Norris Medical Library, University of Southern California, Los Angeles, California, United States of America; 7 Department of Molecular Pharmacology and Toxicology, Keck School of Medicine, University of Southern California, Los Angeles, California, United States of America; EFS, FRANCE

## Abstract

There is currently no FDA approved therapeutic agent for ARS mitigation post radiation exposure. Here we report that the small molecule YH250, which specifically antagonizes p300/catenin interaction, stimulates hematopoiesis in lethally or sublethally irradiated mice. A single administration of YH250 24 hours post irradiation can significantly stimulate HSC proliferation, improve survival and accelerate peripheral blood count recovery. Our studies suggest that promotion of the expansion of the remaining HSC population via stimulation of symmetric non-differentiative proliferation is at least part of the mechanism of action.

## Introduction

Mass casualties due to accidental radiation exposure represent a serious threat to society. Radioprotective agents are partially successful given prior to radiation exposure however, post exposure they have limited utility. This motivated us to search for agents that could alleviate radiation damage post-exposure. Due to logistical considerations, it is preferable to achieve significant radiation mitigation up to 24 h after exposure. Acute radiation syndrome (ARS) after total body exposure to radiation describes an array of symptoms. Fatal injuries, primarily of a hematopoietic nature occur at doses of less than 8Gy [1]. Universal lethality occurs at doses of more than 10Gy due to damage to the gastrointestinal (GI) tract [2]. Current medical countermeasures have limited efficacy and no FDA approved treatment to alleviate ARS or to effectively treat/protect first responders from ARS currently exists.

Expansion of the remaining stem cell population with subsequent utilization of the stem cell pool to regenerate damaged tissues is imperative for successful repair and regeneration after acute radiation injury. Damage to the hematopoietic system, can in principle be alleviated via bone marrow transplantation and supportive care [1], however, this is not possible in the case of the GI tract [2].

Notch, Hedgehog, JAK ⁄ Stat, BMP, Hippo, FGF ⁄ MAPK, and Wnt signaling cooperate to balance self-renewal versus differentiation of adult stem cells [3, 4]. Hematopoietic stem cells (HSCs) are a rare population of somatic stem cells with the ability to regenerate the entire mature blood system in a hierarchical way. The bone marrow niche provides a microenvironment where different cell types and molecules regulate/maintain HSC dormancy or induce the activation of HSC s in both normal and malignant hematopoiesis. In HSCs as well as in other somatic stem cell populations, particularly in intestinal stem cells (ISCs), Wnt signaling plays a critical role [5]. Yet, significant controversy exists regarding whether Wnt signaling is important for proliferation and maintenance of potency (pluripotency or multipotency) or differentiation of stem/progenitor cells [3–8]. We have, for more than a decade, investigated and validated a model of differential coactivator usage that highlights the distinct roles of the coactivators CBP and p300 in Wnt/β-catenin mediated transcription in stem cells. Utilization of the coactivator Kat3A (CBP) or Kat3B (p300) by β-catenin is the first critical decision guiding the stem cell to either proliferate/maintain potency or initiate a differentiative transcriptional program, respectively [9–11]. Our lab has developed specific small molecules that selectively block either the CBP/catenin (e.g. ICG-001) or the p300/catenin interaction (e.g. YH250). CBP/catenin antagonists induce asymmetric differentiation of stem cells [11], whereas p300/catenin antagonists) increase symmetric expansion and maintain potency in stem/progenitor cells *in vitro* [12]. We therefore investigated the therapeutic concept that the p300/catenin antagonist YH250 could remediate radiation damage via symmetric expansion of the remaining viable stem cell pool.

## Results

### P300/catenin antagonist YH250 accelerates hematopoietic recovery in sub- lethally irradiated mice

**Fig 1:**
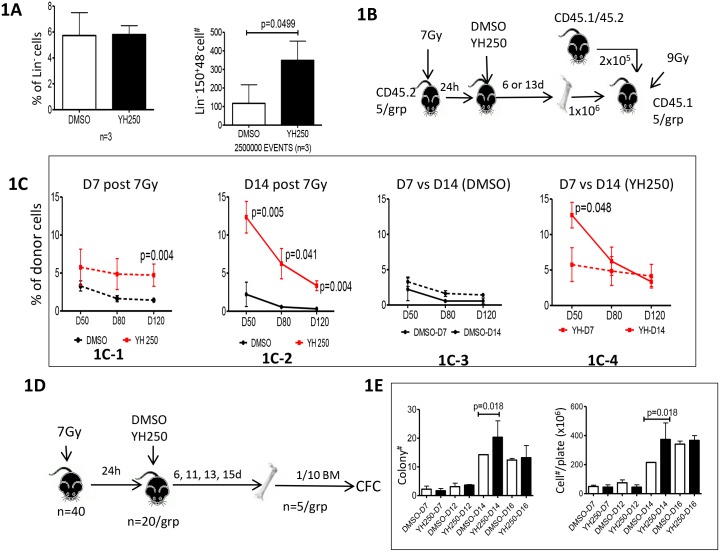
YH250 stimulate hematopoiesis recovery from 7Gy radiation. (A) Bone marrow cells from either YH250 or DMSO treated 7Gy irradiated animals were recovered and FACS analyzed, there are more Lin^-^CD48^-^CD150^+^ cells in YH250 treated animals. (B) At different time post radiation, bone marrow cells were recovered for competitive repopulation or (D) CFC assays. (C) Bone marrow cells recovered at day 14 post radiation from YH250 treated animals give significantly better long-term engraftment in competitive repopulation and (E) more colonies in CFC assay.

We first decided to investigate whether YH250 administration could enhance hematopoietic recovery after sublethal irradiation via expansion of the hematopoietic stem/progenitor population (HSPC). In the event, 24h post 7Gy sub-lethal irradiation, mice were administered YH250 (s.c. 2mg/Kg) or vehicle control and 6h later BrdU was given. The YH250 treated group had a greater percentage of BrdU^+^ cells in the Lin^-^ but not in the Lin^+^ population than control treated mice (S1A Fig). YH250 treatment also increased the percentage of bone marrow cells in S phase, suggesting an increase in cycling activated cells (S1B Fig). The LSK CD150^+^CD48^-^ population represents a “Long Term Repopulating” hematopoietic stem cell (LTR-HSC) population, however the number of these cells is quite limited [13, 14]. Four days after YH250 administration to mice, FACS analysis demonstrated a significant increase in the Lin^-^CD150^+^CD48^-^ population compared with vehicle control, although the overall percentage of Lin- cells was not affected (Fig 1A). Taken together, these results suggest that YH250 treatment can stimulate HSPC proliferation and expansion of the HSPC stem cell pool. However, the Lin^-^ CD150^+^CD48^-^ is still quite heterogeneous, representing a mixed population of HSCs and progenitor cells, therefore functional assays were required to confirm the effect of YH250 on HSPCs.

**Fig 2:**
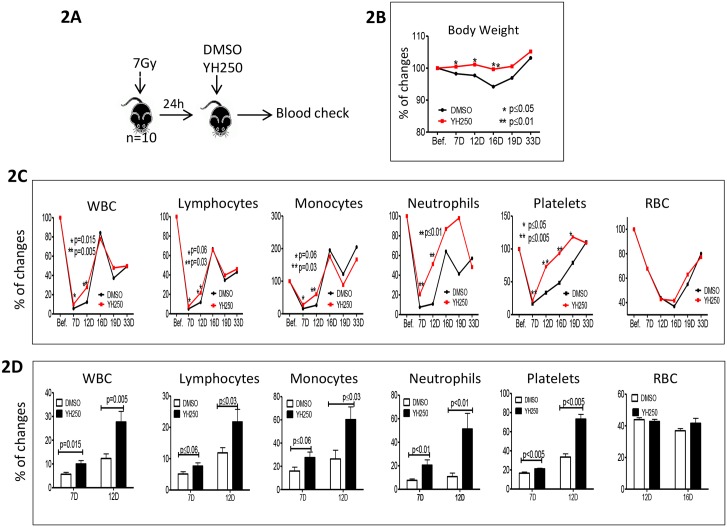
YH250 stimulates multi-linage recovery in peripheral blood of 7Gy irradiated animals. (A) The experimental procedure to test YH250 effect to peripheral blood recovery in 7Gy irradiated animals is depicted. (B) The body weight change of the animals is shown. (C) Peripheral blood counts were monitored. (D) The blood counts at nadir points are presented. *: p<0.05; **: p<0.01. n = 10.

**Fig 3:**
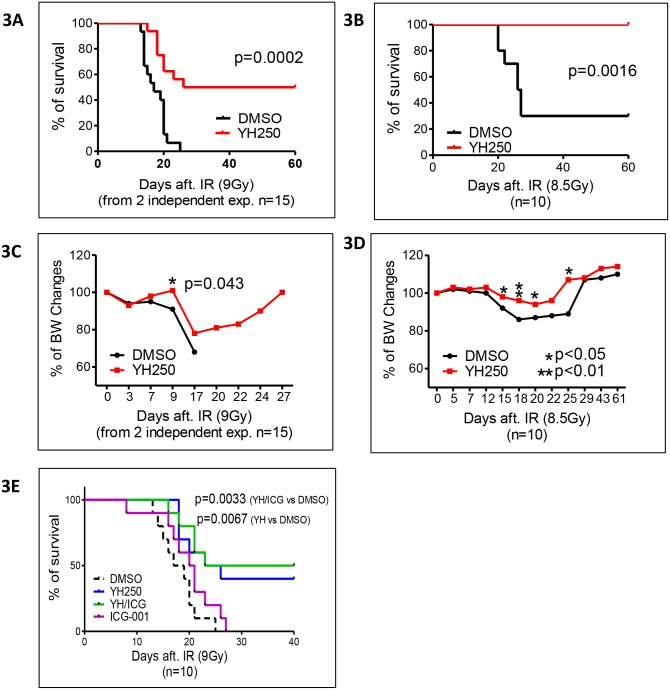
YH250 rescues animals from lethal dose radiation. (A and B) After lethal dose radiation (either 9Gy or 8.5Gy), animal survival and body weight (C, D) were monitored. (E) The combination effect of YH250 with ICG-001 to lethally irradiated animals. *: p<0.05; **: p<0.01.

**Fig 4:**
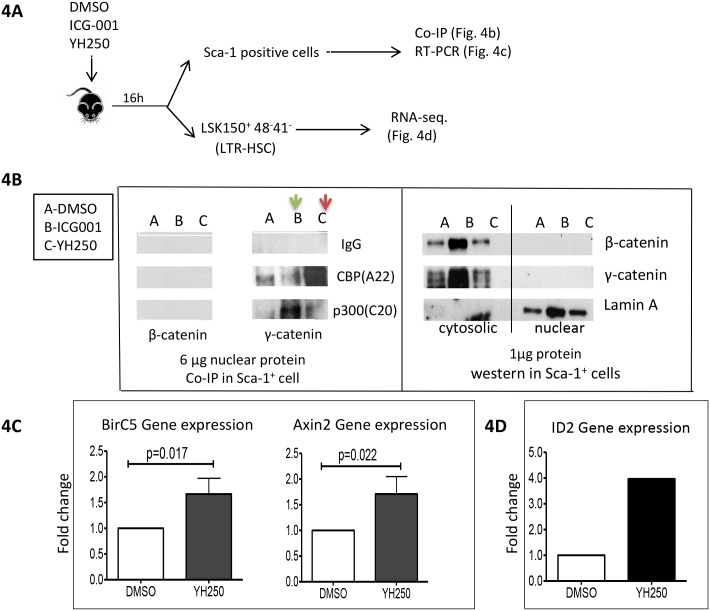
YH250 biochemical mechanism and its downstream gene expression regulation. (A) The procedures to isolate Sca-1^+^ cells (for assays in 4B and 4C) or LSK150^+^41^-^48^-^ cells (for assay in fig 4D) are depicted. (B) Left panel: CO-IP analysis to detect the interaction of catenin with CBP or p300 were depicted: the green arrow shows the sample from ICG-001 treated animals, the red arrow shows the sample from YH250 treated animals. Right panel: western blot analysis of β- and γ-catenin in Sca-1^+^ cells. (C) qPCR analysis results were summary of 3 independent experiments. (D) Id2 gene up-regulation from YH250 treated animal LSK150^+^ 41^-^48^-^ cells from RNA—seq analysis.

In the hematopoietic system, the LTR-HSC or pluripotent HSC sits atop the hematopoietic hierarchy. LTR-HSCs subsequently generate “Short Term Repopulating” hematopoietic stem cells (STR-HSC), and then progenitor cells [15–17]. After radiation induced myeloid-ablation, there is a significant loss of progenitor cells. To demonstrate enhanced hematopoietic recovery we evaluated whether YH250 treatment provided for the earlier detection of cells with more mature functionality. Bone marrow cells were recovered at different time points from YH250 or vehicle treated 7Gy irradiated mice to test for HSPC functionality in competitive repopulation assays (Fig 1B) and CFC assays (Fig 1D), which can be used to detect LTR-HSC/STR-HSC or progenitor cell activity, respectively. In the competitive BMT repopulation study, bone marrow cells from YH250 treated animals, recovered at day 7 or day 14 post irradiation, gave significantly higher engraftment compared with controls (Fig 1C-1 to 1C-4). It is also worth noting that cells recovered from YH250 treated animals at day 14 post irradiation gave significantly higher engraftment at an earlier stage (day 50 post BMT), compared with cells recovered from day 7 post irradiation. However, there was no significant difference in long term engraftment (day 120 post BMT) between the cells recovered at day 7 and day 14(Fig 1C-4). Since engraftment capability at day 50 and 120 post BMT represent the effects of the STR-HSC and LTR-HSC respectively, this result suggests that YH250 stimulated LTR-HSC expansion occurred within the first 6 days after administration. The expanded LTR-HSC population proliferates and matures into STR-HSC by day 14, thereby providing enhanced short term engraftment. There apparently is no further LTR-HSC expansion from day 6 to day 13 post YH250 administration. This can explain why cells recovered from day 14 show dramatically increased engraftment at day 50, yet similar engraftment at day 120 compared with cells recovered at day 7 (Fig 1C-4). CFC assay also demonstrated increased colony formation from cells recovered at day 14 post irradiation in the YH250 group (Fig 1E). This suggests that more progenitor cells were produced in the YH250 treated animals by day 14. Overall, this suggests that YH250 initially stimulates HSPC expansion thereby accelerating hematopoietic recovery in sub-lethally irradiated mice.

We next evaluated whether YH250 could accelerate blood count recovery (Fig 2A). Administration of a single dose of YH250, 24h post irradiation, accelerated multi-lineage hematopoietic recovery (Fig 2C). At the nadir point, YH250 treated mice demonstrated significantly higher blood counts, including WBC, lymphocytes, monocytes, neutrophils, and platelets (Fig 2D). Additionally, mice that received YH250 demonstrated significantly less loss of body weight (Fig 2B). Taken together, these results demonstrate the ability of YH250 to mitigate sub-lethal irradiation induced myelosuppression by initially stimulating LTR-HSC expansion *in vivo*.

### P300/catenin antagonist YH250 rescues mice from lethal dose radiation

Next, we tested whether YH250 can rescue mice from lethal dose radiation. Mice received 9Gy (LD_100_) or 8.5Gy (LD_70_) whole body radiation. 24h later, vehicle control or YH250 (2mg/Kg) was administered subcutaneously. All mice in the control group died within 30 days post 9Gy irradiation. However, YH250 administration 24 h post irradiation rescued 50% of the mice (Kaplan-Meier analysis p = 0.0002) (Fig 3A). After 8.5Gy irradiation, 100% of the mice in the YH250 group survived, whereas only 30% of the vehicle control mice survived (p = 0.0016) (Fig 3B). We also observed significantly less loss of body weight after irradiation in the YH250 treated mice at both 9Gy and 8.5Gy (Fig 3C and 3D).

### Combination of CBP/catenin and p300/catenin antagonists in radiation mitigation

In principle, to optimize radiation remediation therapy, it should be advantageous to first (a) symmetrically expand the remaining viable stem cell pool (either HSC or ISC), using a method to enhance symmetric non-differentiative proliferation, i.e. by blocking the p300/catenin interaction thereby enhancing CBP/catenin signaling and (b) subsequently induce the differentiation of the stem cell pool via asymmetric divisions to enhance tissue repair and regeneration as rapidly as possible using a CBP/catenin antagonist. We therefore decide to test sequential administration of the p300/catenin antagonist YH250, followed by subsequent administration of the CBP/catenin antagonist ICG-001. An additional potential therapeutic benefit utilizing a CBP/catenin antagonist would be protection against fibrosis, which is a common chronic complication associated with radiation damage. CBP/catenin antagonists have previously demonstrated efficacy in multiple pre-clinical models of fibrosis in lung, kidney, liver etc. [18–21]. In this experiment, mice were treated with either vehicle, YH250 (single 2mg/Kg s.c. injection at 24 h post irradiation), ICG-001 (single 50mg/Kg s.c injection at 24 h post irradiation) or the combination of YH250/ICG-001, with YH250 (2mg/Kg) given 24h post irradiation followed by ICG-001(50mg/Kg) injection for 5 consecutive days starting at 48h post-irradiation. After irradiation and the corresponding treatments, no special care was provided. The life-span after irradiation was observed on a daily basis and recorded. The survival curves are shown in Fig 3E. Mice that received either YH250 or a combination of YH250 and ICG-001 showed significantly extended life-spans (p = 0.0067 and 0.0033 for the YH 250 and YH250/ICG-001 groups versus the control group respectively).

### Potential mechanism of action of YH250

To begin to investigate the biological mechanism of action that provides the p300/catenin antagonist YH250 with the capacity to enhance hematopoietic recovery after irradiation, we undertook a series of investigations. It should be noted, that while some of the effects of YH250 are HSC intrinsic, YH250 likely has effects on the bone marrow microenvironment, i.e. the HSC niche, which we have not investigated, yet may also contribute to its *in vivo* efficacy. In the event, we used mouse bone marrow Sca-1^+^ cells, which are enriched for LTR-HSCs, STR-HSCs, progenitor cells as well as other cell types required for blood production and the bone marrow microenvironment. 16 hours after YH250 or vehicle administration, Sca-1^+^ cells were isolated for co-IP analysis (Fig 4A). From 6μg of nuclear protein fraction, β-catenin could not be detected in either the CBP or p300 immunoprecipitates. However, γ-catenin was found, bound principally to CBP and to a lesser extent p300. As shown in Fig 4B (left panel), the CBP/catenin antagonist ICG-001, blocked the CBP/γ-catenin interaction thereby enhancing the p300/γ-catenin interaction (lane B, green arrow). As anticipated, YH250 had the opposite effect, increasing the CBP/γ-catenin interaction at the expense of the p300/γ-catenin interaction (lane C, red arrow). This confirms that YH250, as previously reported for other cell types [12] directly disrupts the p300/catenin interaction thereby enhancing the CBP/catenin interaction in HSPCs. We also examined the expression of β-catenin in Sca-1+ cells. Utilizing 1μg of total protein from these cells, both β- and γ-catenin were readily detected in the cytosolic fraction but not in the nuclear fraction (4B, right panel). This suggests that the majority of both β- and γ-catenin is cytosolic in the Sca-1+ population under normal physiological conditions (Fig 4B, right panel). To confirm the specificity of the antibodies utilized in these experiments, we chose the human lung carcinoma cell line NCI-H28, which expresses γ-catenin, however does not express β-catenin due to a chromosomal deletion, and SW480 human colorectal carcinoma cells, which express both proteins (S2 Fig).

We next investigated Wnt/catenin downstream target gene expression after YH250 administration. Axin2 is a classical Wnt/catenin target genes and plays important regulatory roles in Wnt signaling [22]. The inhibitor of apoptosis (IAP) family member survivin (*Birc5*) is another Wnt/catenin target gene [23]. We have previously demonstrated that ICG-001, by antagonizing the CBP/catenin interaction, down-regulates *survivin* expression in a variety of cell types both *in vitro* and *in vivo* [24–26]. As shown in Fig 4C, both *Axin2* and *survivin* expression were significantly up-regulated by YH250 treatment in the Sca-1^+^ population. Next, we treated mice with either vehicle or YH250 and16h later collected the LTR-HSC (LSK CD150^+^CD48^-^CD41^-^) and the progenitor (LSK CD150^+^CD41^+^CD48^+^) populations by FACS. RNA was isolated from these two populations and RNA-seq was performed. We subsequently used Partek E/M to analyze the genes that were differentially regulated by treatment with YH250 in the LTR-HSC (LSK CD150^+^CD48^-^CD41^-^) population. Interestingly, one of the most significantly upregulated genes in the LTR-HSC but not in the progenitor population was Inhibitor of DNA Binding 2 (Id2). Its expression was up-regulated 4 fold in the YH250 treated samples (Fig 4D). Id2 is an HLH protein Wnt/catenin target gene [27], which plays pivotal roles in stem cell self-renewal and maintenance. It has been previously reported that elevated Id2 expression correlates with CD34^+^ HSC non-differentiative proliferation and results in CD34^+^ HSC expansion [28]. Also, HSCs with higher Id2 expression show better engraftment in competitive repopulation assays [29]. Other genes that were significantly upregulated in the LTR-HSC and not in the progenitor population included CD52 (3.09 fold), a membrane protein that is found on the surface of HSPCs [30], and the tetraspanin protein CD53 (2.79 fold), which is associated with the CD34^+^CD133^+^ cord blood HSC population [31](S1 Table). Based upon these results, we investigated the ability of YH250 to promote HSC non-differentiative proliferative divisions *in vitro* and *in vivo*.

**Fig 5:**
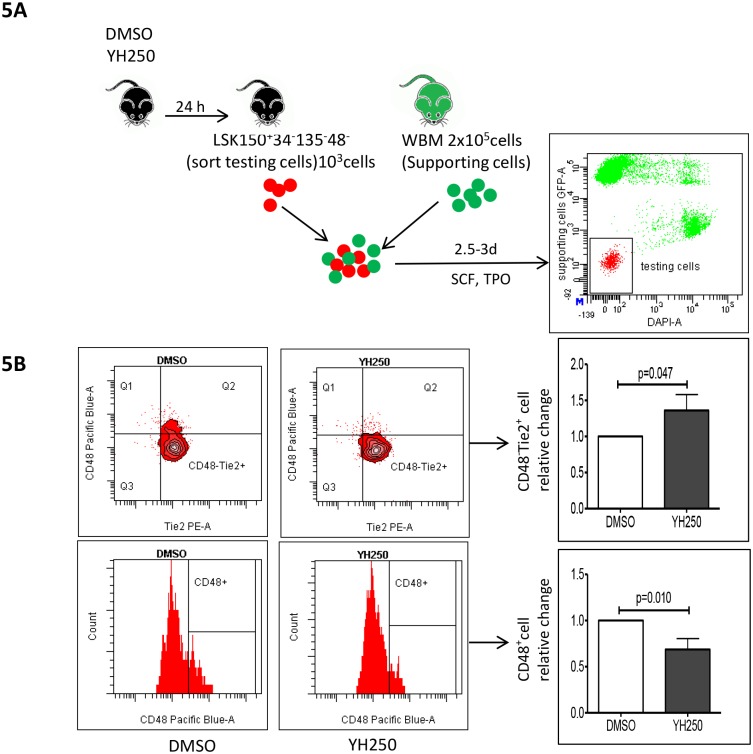
YH250 sustains LT—HSC in vitro. (A) LSK34^-^135^-^150^+^ 48^-^ cell preparation is depicted. (B) Cell surface marker changes from DMSO or YH250 treated animals in in vitro culture. Results are summary of 3 independent experiments.

It has been previously reported that LSK34^-^135^-^150^+^48^-^ cells that maintain the CD48^-^Tie2^+^ phenotype under *in vitro* culture conditions retain LTR-HSC capacity, whereas cells that become CD48^+^ lose their LTR-HSC activity [32–33]. Mice were administered vehicle or YH250 (2mg/Kg), 16h prior to sacrifice and subsequent FACS sorting of bone marrow cells. Sorted LSK34^-^135^-^150^+^48^-^ cells were subsequently co-cultured with whole bone marrow cells from GFP transgenic mice for 2 days (Fig 5A). Cells were then recovered from the culture and analyzed for CD48 and Tie2 surface expression. Compared to vehicle control treated animals, there is a significant increase in the percentage of CD48^-^Tie2^+^ cells in YH250 treated mice (Fig 5B).

Next, we tested if brief incubation of YH250 with bone marrow cells in an *in vitro* culture system affects stem/progenitor cell activity. YH250 was incubated with bone marrow cells for 4hrs and subsequently washed out, before the cells were subjected to colony forming cell (CFC) assay. There was no significant difference between DMSO and YH250 treated cells in regards to the number of colonies formed (S3A Fig). However, we consistently observed that the colony size from YH250 treated cells was larger than vehicle treated cells (S3B Fig). We then recovered the cells from each plate for counting. YH250 treatment consistently generated significantly higher cell numbers in the CFC assay (S3C Fig). Next, we injected YH250 treated cells into lethally irradiated animals and performed a CFU-S_12_ assay. We again observed similar effects in that YH250 treatment provided larger colonies (S3E Fig) and increased spleen weight (S3F Fig) although there was no significant difference in colony number (S3D Fig). Taken together, these results suggest that YH250 treatment provided greater cell expansion and an increase in the maintenance of the LSK34^-^135^-^150^+^48^-^ population via enhancing symmetric cell division, thereby retaining the LT-HSC phenotype.

### YH250 stimulates HSC proliferation

**Fig 6:**
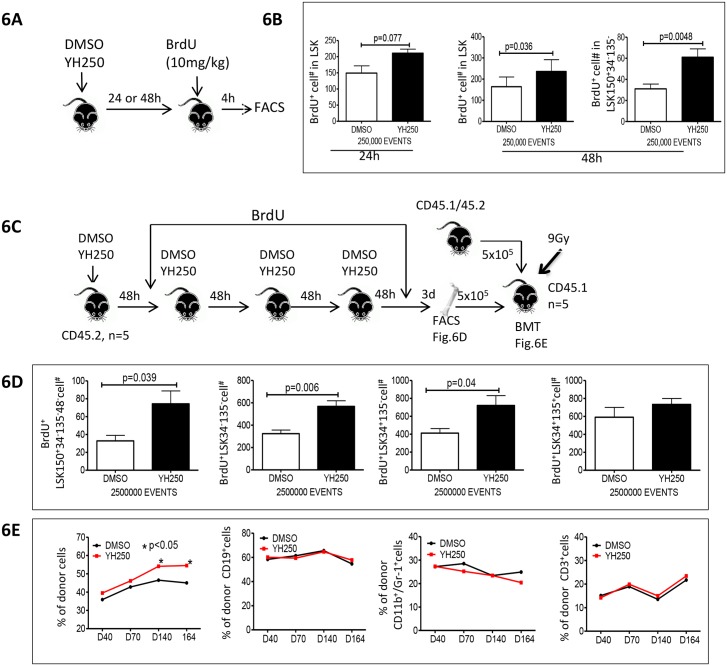
YH250 stimulates HSPC non-differentiation proliferation in vivo. **(A)** BrdU incorporation studies were performed as depicted with 3 mice in each group and two independent experiments. (B) BrdU^+^ cells in BM subsets. (C) Experimental procedure to study repeated YH250 administration effects on HSPC in steady status was depicted. (D) At the end of last YH250 or DMSO administration, bone marrow cells FACS analysis and (E) competitive repopulation assay.

To study the effects of YH250 on HSPC proliferation, we examined BrdU incorporation. Our initial investigations were performed in mice under steady-state conditions. 24 or 48 h post YH250 or vehicle administration, BrdU was administered 4h prior to sacrifice and isolation of bone marrow cells (Fig 6A). As shown in Fig 6B, there was a significant increase in the BrdU^+^ cells in both the LSK and LSK34^-^135^-^150^+^ populations 48 h post YH250 administration. This suggests that YH250 stimulated LTR-HSC proliferation. We hypothesized that if YH250 stimulated LTR-HSC symmetric non-differentiative proliferation, we should be able to detect the expansion of the LTR-HSC stem cell pool via competitive repopulation assay. To test this hypothesis, we administered YH250, 4 times at 48h intervals, while BrdU was supplied in the drinking water. Three days after the final YH250 administration, bone marrow cells were isolated for FACS analysis and competitive repopulation assay (Fig 6C). There was a significant increase in the number of BrdU^+^ cells in the LTR/STR-HSC population with YH250 treatment (Fig 6D). In contrast, there was no statistically difference in the MPP population (BrdU^+^LSK34^+^135^+^) between the YH250 treated and control mice.

We then mixed YH250 or vehicle treated mouse bone marrow cells with competitor cells to perform a competitive repopulation assay, a gold standard for examination of LTR-HSC capacity. As depicted (Fig 6E), there was no significant difference in terms of short term engraftment (day 40 post BMT) amongst the two groups. However, YH250 treatment provided significantly higher long-term engraftment (day 140 and 164 post BMT) compared with controls, without any significant lineage biasing (Fig 6E). These results suggest YH250 can stimulate LTR-HSC symmetric non-differentiative proliferation under steady-state hematopoiesis.

## Discussion

Accidental radiation exposure presents a serious threat and existing medical countermeasures are quite limited and no FDA approved treatment is currently available to treat victims or first responders from ARS. Expansion and utilization of the remaining stem cell pool to regenerate damaged tissues is critical for repair and regeneration after acute radiation injury. In the current study, we were motivated to search for stratagems to alleviate post-exposure radiation damage. We utilized the small molecule YH250, which specifically antagonizes the p300/catenin interaction that we had previously shown to maintain pluripotency in both mouse and human cells (12). We first demonstrated that YH250 enhances hematopoietic recovery after sublethal irradiation. Administration of a single dose of YH250, 24h post irradiation, accelerated multi-lineage hematopoietic recovery and at the nadir point, YH250 significantly increased blood counts. Additionally, mice receiving YH250 demonstrated significantly less body weight loss. YH250 treatment 24h post irradiation also substantially increased survival after lethal radiation.

To begin to investigate the mechanism of action that endows the p300/catenin antagonist YH250 with the capacity to enhance hematopoietic recovery, we undertook a series of investigations on the HSC intrinsic effects of YH250. In mouse bone marrow Sca-1^+^ cells, which includes the LTR-HSC population, YH250 increased the CBP/γ-catenin interaction at the expense of the p300/γ-catenin interaction. In our co-IPs most of the detectable coactivator-bound catenin was γ- and not β-catenin (Fig 4B). Based on these results, we propose that γ-catenin rather than β-catenin is the primary driver of Wnt/catenin signaling in mouse HSPCs. It is been previously documented that β- and γ-catenin share similar biological functions and although not redundant, they can compensate for each other in a variety of systems [34–36]. Their functions in HSC and cancer stem cells have been investigated, although most studies to date have focused on β-catenin [3–8,37–39]. The Kat3 transcriptional coactivators CBP and p300 play pivotal roles in stem cell self-renewal and/or differentiation and both CBP and p300 interact with β- and gamma catenin to regulate gene expression [40–42]. Based upon our results, we caution that previous “gain or loss of function” studies with β-catenin may not accurately reflect normal physiological conditions in HSPCs. Interestingly however, even double deletion of β- and γ-catenin did not affect LTR-HSC activity and lymphopoiesis, thereby pointing to an as yet identified alternative catenin-like molecule that can compensate in hematopoiesis under these conditions [43–44].

Sorted LTR-HSC (LSK34^-^135^-^150^+^48^-^ cells) from mice administered YH250 16h prior to sacrifice demonstrated a significant increase in the percentage of CD48^-^Tie2^+^ cells upon *ex vivo* expansion. YH250 treatments although not affecting colony number, consistently increased colony size. Taken together, our results demonstrate that YH250 can stimulate LTR-HSC symmetric non-differentiative proliferation enhancing hematopoiesis.

However, there are a number of limitations to our mechanistic studies that should be considered: 1) Due to limited cell numbers, we could not perform co-IP experiments with pure LTR-HSC. Sca-1^+^ cells represent a mixed cell population including HSPC as well as other cell types, i.e. mesenchymal and stromal cells; 2) the amount of protein used in these co-IP experiments was significantly less than we normally use (6μg of instead of 150–200 μg for co-IP; and 1 μg total protein instead of 10 μg for immunoblots); 3) while some of the effects of YH250 are HSC intrinsic, YH250 likely has effects on the bone marrow microenvironment as well, which we did not investigate. However, they may make important contributions to YH250’s *in vivo* efficacy since Wnt signaling also play important roles in HSC niche [45]. Nevertheless, our result suggests γ-catenin plays important roles in HSCs, under both physiological and pathophysiological conditions. YH250 in HSCs directly disrupts the p300/γ-catenin interaction thereby enhancing the CBP/γ-catenin interaction, and increasing the expression of a subset of Wnt/catenin target genes (e.g *survivin/bir*c5, *id2* and *axin2*)[46–47]. The differential effects of CBP (HSC self-renewal) and p300 (hematopoietic differentiation) in hematopoiesis have been previously reported [41, 48]. Id2 has been reported to play important roles in HSC biology, maintaining stem cell pluripotency and self-renewal capacity. Forced expression of Id2 in human CD34^+^ cells results in non-differentiative proliferation and stem cell expansion [28]. In the present study, YH 250 demonstrated HSC expansion in both the steady state and during myeloid suppression after irradiation, coinciding with increased Id2 expression. Survivin/birc5 is known for its anti-apoptotic and proliferation-stimulating effects. YH250, up-regulated *survivi*n gene expression in the Sca-1^+^ population after irradiation, thereby presumably enhancing hematopoietic recovery. YH250 treated mice also demonstrated less body weight loss after radiation (Figs 2B, 3C and 3D). This phenomenon may be correlated with the decreased nadir blood count and enhanced rapidity of hematopoietic recovery after YH250 treatment.

In order to optimize hematopoietic recovery after irradiation, in principal initial symmetric expansion of the viable HSCs via symmetric non-differentiative proliferation, with subsequent induced differentiation of the expanded stem cell pool via asymmetric divisions, would be ideal. We therefore tested sequential administration of the p300/catenin antagonist YH250, followed by subsequent administration of the CBP/catenin antagonist ICG-001. Mice that received the combination of YH250 and ICG-001 demonstrated a somewhat enhanced life-span extension in our studies compared to YH250 treatment alone.

Wnt/catenin signaling has been shown to be important in hematopoiesis. Previously, other modulators of Wnt signaling, including PGE2, GSK3 inhibitors as well as others, have been shown to increase murine long term HSC populations *in vivo* [49–50]. Here, we demonstrate that the small molecule p300/catenin antagonist YH250 also increases murine long term HSCs *in vivo* by promoting the CBP/catenin interaction. Further investigation combining p300/catenin antagonists with other modes of hematopoietic stimulation will hopefully provide a deeper understanding of hematopoietic regulation.

## Materials and methods

### Animal studies, CFC and CFU-S12 assays

All animal studies were approved by the USC institutional IACUC committee. C57BL/CD45.1, CD45.2 or GFP transgenic mice were purchased from Jackson Laboratory. CD45.1 and CD45.2 hybrid were bred at the University of Southern California animal’s facility. Female mice at age 8–10 weeks were used in the study. Animals were random assigned to each treatment group with simple randomization. Investigators were not blind in animal study. After whole body irradiation, the animals were given soft food and hydrogel for nutrition and hydration (BioServ Product # S3472, Flemington, NJ 08822, USA and Clear H2O, Cat# 70-01-5022, Westbrook, ME, 04092, USA). Fresh soft food and hydrogel were given daily to replace any leftover from the previous day. After receiving whole body irradiation, the irradiated animals were observed twice daily including assessment of behavior, weight and hydration. Following humane end points were leading to immediate euthanasia: animals that were moribund or unable to move or failure to respond to gentle stimuli; labored breath, particularly if accompanied by nasal discharge and/or cyanosis; inability to eat or drink; diarrhea and incontinence continue beyond 72 hrs provided the animals exhibiting signs of dehydration; weight loss above 20% of body weight; spontaneous bleeding and severe clinical distress. The following criteria were used as signs of dehydration and as a humane end-point: skin tenting, sunken eyes and weight loss. A staff veterinarian consulted on ambiguous cases. Animals were euthanized using a CO_2_ chamber and cervical dislocation was performed on each animal after the CO_2_ chamber. If we observed animals in pain, buprenorphine was given to animals twice daily subcutaneously for 48 hrs. There was no unexpected animal death in the survival study. All the death in the survival study occurred 10 days post radiation, at that time the blood cell count decreased to nadir levels, suggesting that death was associated with severe bone marrow suppression.

For in vitro HSC culture assay, sorted HSC were mixed with whole bone marrow cells from GFP mice and cultured in QBSF-58 medium (Serum free medium for support of murine bone marrow cells, Quality Biological, Inc. Cat# 160-109-101) supplemented with TPO and SCF (10ng/ml for each, R & D systems, Cat#P40229 and Cat#Q78ED8, respectively). CFC and CFU-S12 assays were performed as described previously [51]. In BrdU study, mice were given either 1mg/mouse i.p or 0.8mg/ml in drinking water.

For irradiation study, mice were subjected to 7 or 8.5 or 9 Gy irradiation by placing in X-rad 320i irradiator (Precision X-ray, Inc.). In competitive repopulation assays, testing bone marrow cells were mixed with competitor cells and injected to 9Gy irradiated recipients via tail vein. Blood were collected via submandibular vein and analyzed with Hemavet (Drew Scientific) for blood count.

### Immunoprecipitation and immunoblotting

Immunoprecipitation and imunoblotting were performed as described previously [26]. The reagents were as following: anti-β-catenin (BD Bioscience, Cat# 610153), anti-γ-catenin (BD Bioscience, Cat# 610253), anti-CBP (Santa Cruz, clone SC-369), anti-p300 (Santa Cruz, clone SC-584), anti-activated- β-catenin (Millipore, clone 8E7), anti-lamin A/C (Santa Cruz, SC-7293), NE-PER Nuclear extraction reagent (Pierce, Cat#78833), Protease inhibitor cocktail (Calbiochem, Cat#539137), Protein A-agarose (Roche, Cat#11134515001), Illustra microspin columns (GE Healthcare, Cat#27-3565-01), ECL Plus (GE Healthcare, Cat#RPN 2132), and Blue ultra autorad film (BioExpress, Cat# F9029-8X10).

### Gene expression study

Total RNA was isolated, cDNA synthesis and q-PCR were performed as previously described [26] using the following primers: Axin 2: F 5’-GAGAGATGCATCGCAGTGTG, R 5’-AAGGCAGCAGGTTCCACAG; survivin: F 5’-TACCGAGAACGAGCCTGATT, R 5’-CCAGGGGAGTGCTTTCTATG; ID2: F 5’-ACTATCGTCAGCCTGCATCA, R 5’-ATTCAGATGCCTGCAAGGAC; GUS-B F 5’-AGAATACGTGGTCGGAGAGC, R 5’-CGACTGAAGATCCCCTTCTT.

Libraries were generated and sequencing was performed at the USC Epigenome Center in the Norris Comprehensive Cancer Center. Briefly, total RNA was prepared from the sorted cell fractions and amplified cDNA generated using SMARTer Ultra Low Input RNA for Sequencing-v3 kit (Clontech Laboratories) according to manufacturer’s instructions. The amplified cDNA was subsequently analyzed on the Bioanalyzer (Agilent), then sonicated to appropriate size (300–500 bp) using a Covaris S2 sonicator. DNA fragments were made into libraries using the KAPA DNA Library Preparation Kit (KAPA Biosystems) for Illumina sequencing platforms, using minimal number of PCR cycles to insure complexity. Libraries were applied to an Illumina flow cell and sequenced on Illumina HiSeq 2000 to generate 50 bp single end reads. Total reads per sample averaged around 31 million. Image analysis and base calling was carried out using RTA 1.13.48.0. Final file formatting, demultiplexing and fastq generation were carried out using CASAVA v 1.8.2.

RNA-seq data was analyzed with Partek Flow version 4 (Partek Inc., St. Louis, MO). Raw sequencing reads were first trimmed from both ends with Quality Score method (bases with quality score Phred less than 20 were trimmed from both ends, and trimmed reads shorter than 25 nt were excluded from downstream analyses). Trimmed reads were then mapped to the mouse genome mm10 using Tophat version 2.0.8 (Kim et al. 2013) with default parameter settings and using Gencode M3 annotation (Mudge JM and Harrow J 2015) as guidance. Gencode M3 annotation was used to quantify the aligned reads to genes/transcripts using Partek E/M method. Finally, read counts per gene/transcript in all samples were normalized using Upper Quartile normalization (Bullard et al. 2010) and analyzed for differential expression using Partek Gene Specific Analysis method (genes/transcripts with less than 10 reads in any sample among a data set were excluded). The differentially expressed gene (DEG) lists were generated for each comparison using the cutoff of FDR<0.05 and fold changes greater than 2 either direction. Subsequent functional analysis of the DEG lists were carried out using Ingenuity Pathway System (Qiagen, Redwood City CA).

### FACS analysis and cell sorting

BD LSRFortessa or BD Aria II flow cytometer were used for sample analysis or sorting respectively. Antibodies and isotype controls for immunostaining were purchased from eBioscience (San Diego, CA, USA): FITC lineage: Gr-1 1 (Ly-6G/C) clone RB6-8C5, Cat#11-5931-82, CD3e clone 145-2C11, Cat #11-0031-82, Ter119 clone TER-119, Cat #11-5921-82, CD11b clone M1/70, Cat #11-0112-85, and CD 45R (B220) clone RA3-6B2, Cat #11-0112-85). Other antibodies used were CD3(clone 145-2c11, PE-cy7 Cat #25–0031), B220 (clone RA3-6B2, APC-cy9 Cat #47–0452) Ter119 (clone Ter-119, PE-cy7 Cat #25–5921, percp-cy5 Cat #45–5921), CD45.1 (clone A20, percp-cy5.5, Cat #45-053-80 and PE Cat #12–0453), CD45.2 (clone 104, FITC Cat #11–0454, percp-cy5.5 catalog 45–0454, e450 Cat #48–0454); CD48 (clone HM48.1, e450 Cat #48-0481-82), CD41 (clone eBIOMWReg30, e450 Cat #48-0411-82), CD150 (clone mShad150, perCp-efluor 710, Cat #46-1502-82), CD117 (kit, clone 288, APC-efluor 780 Cat 47-1171-82, PE Cat #12–1171), Sca1 (clone D7, APC Cat #17-5981-81, percp-cy5.5 Cat #45–5981), CD34 (clone RAM34, Alexa efluor 700 Cat #56-0341-82, APC Cat #56–0341, e450 Cat #48–0341), and CD135 (, clone A2F10, PE-Cy7 Cat #15-1351-82), CD202b (Tie2, clone TEK4, PE Cat #12-5987-83). Lineage Depletion Kit (Cat #130-090-858) and anti-Sca-1 microbead kit are from Miltenyi Biotec GmbH (Cambridge, MA, USA). BrdU flow kit (Cat#559619) is from BD Bioscience.

### Chemicals and synthesis of YH250

5-Bromo-2’-deoxyruidine (BrdU, Cat # B5002) and DAPI (Cat # D9564) are from Sigma-Aldrich (St. Louis, MO, USA). YH250 were synthesized in house as described previously [12].

### Statistical analysis

Two-tailed, unpaired student’s t test or log-rank (Kaplan-Meier) test was performed using GraphPad Prism 5. Data were expressed as mean ±SD.

## Supporting information

S1 Fig YH250 stimulates HSPC proliferation after 7Gy radiation. (A) Under 7Gy radiation, YH250 treated animals show more BrdU incorporation in bone marrow Lin- population and (B) more cells are into cell cycle.
(TIF)

S2 Fig Confirm antibodies used in biochemical studies. Antibodies used in CO-IP (Fig 4) were confirmed in cells which lack β-catenin but express γ-catenin (NCI-H28) or cells which express both (SW480). The red arrow pointed at bands that might be catenin-like protein in both cell lines detected with antibody to activated β-catenin. The green arrow shows the activated β-catenin.
(TIF)

S3 Fig YH250 stimulates HSPC proliferation activity in *in vitro* culture. (A-C) CFC or (D-F) CFU-S_12_ assay with bone marrow cells treated with either DMSO or YH250 for 4 hours in vitro. Results shown are represents from 3 independent experiments.
(TIF)

S1 Table RNA-seq analysis of gene differential expression in LSK150^+^48^-^41^-^ cells from YH250 vs DMSO treated animals. (DOC)
