# Supplementary figures and images for: Small molecule p300/catenin antagonist enhances hematopoietic recovery after radiation
Source: PLoS One. 2017 May 9;12(5):e0177245. doi: 10.1371/journal.pone.0177245 (PMC5423697; doi:10.1371/journal.pone.0177245)

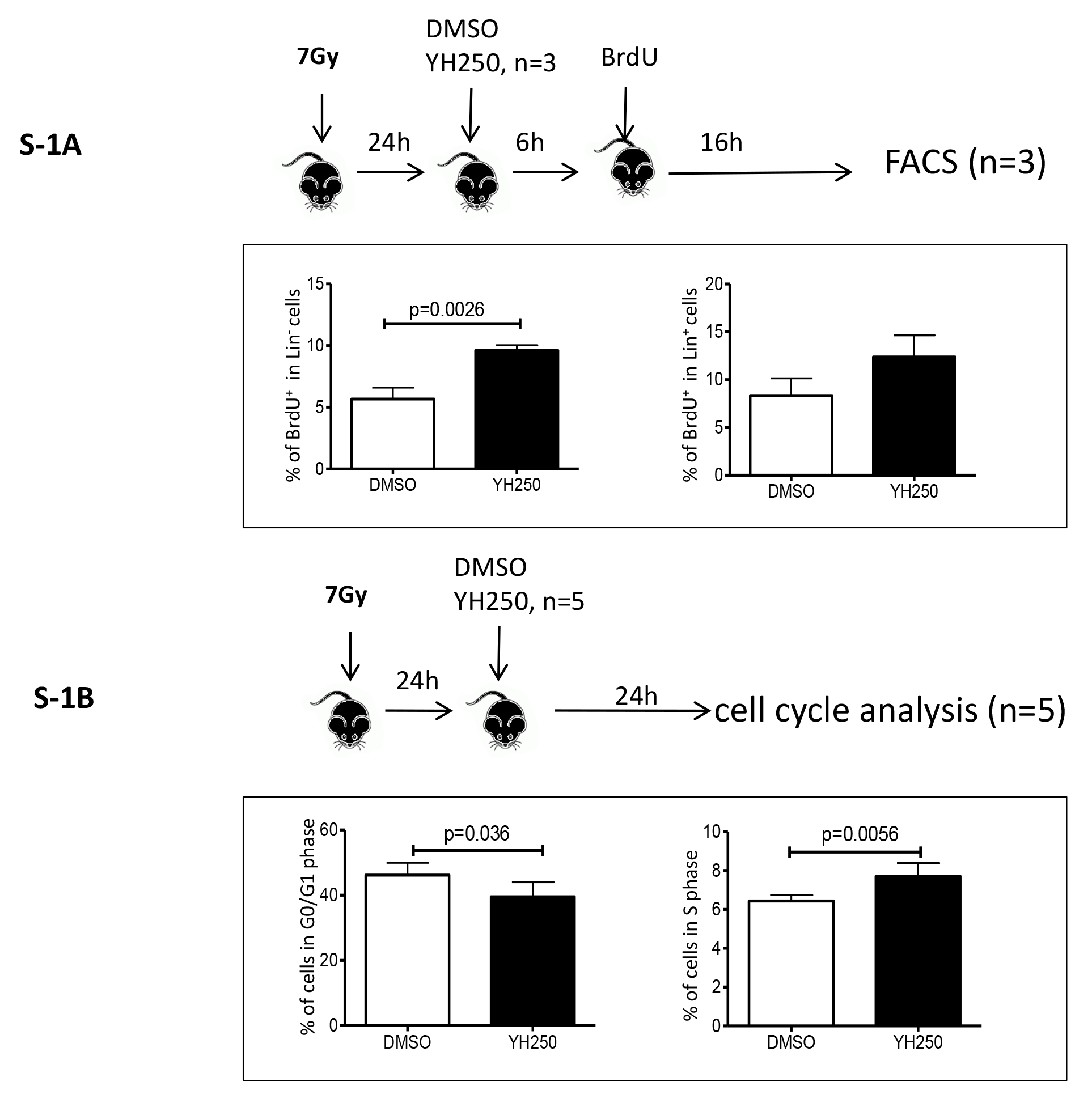

Supplement: S1 Fig — (A) Under 7Gy radiation, YH250 treated animals show more BrdU incorporation in bone marrow Lin- population and (B) more cells are into cell cycle. (TIF) [file pone.0177245.s001.tif]

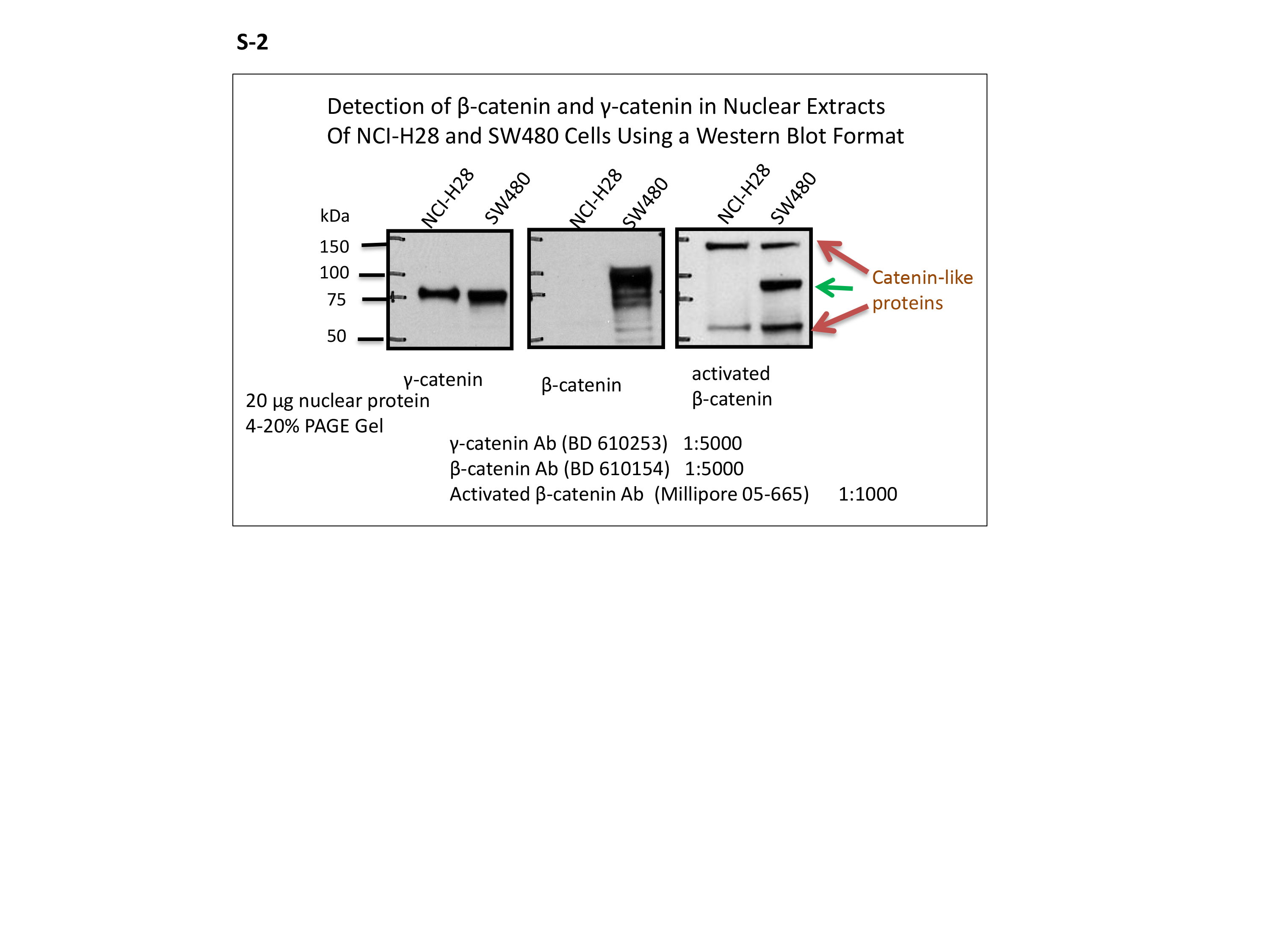

Supplement: S2 Fig — Antibodies used in CO-IP (Fig 4) were confirmed in cells which lack β-catenin but express γ-catenin (NCI-H28) or cells which express both (SW480). The red arrow pointed at bands that might be catenin-like protein in both cell lines detected with antibody to activated β-catenin. The green arrow shows the activated β-catenin. (TIF) [file pone.0177245.s002.tif]

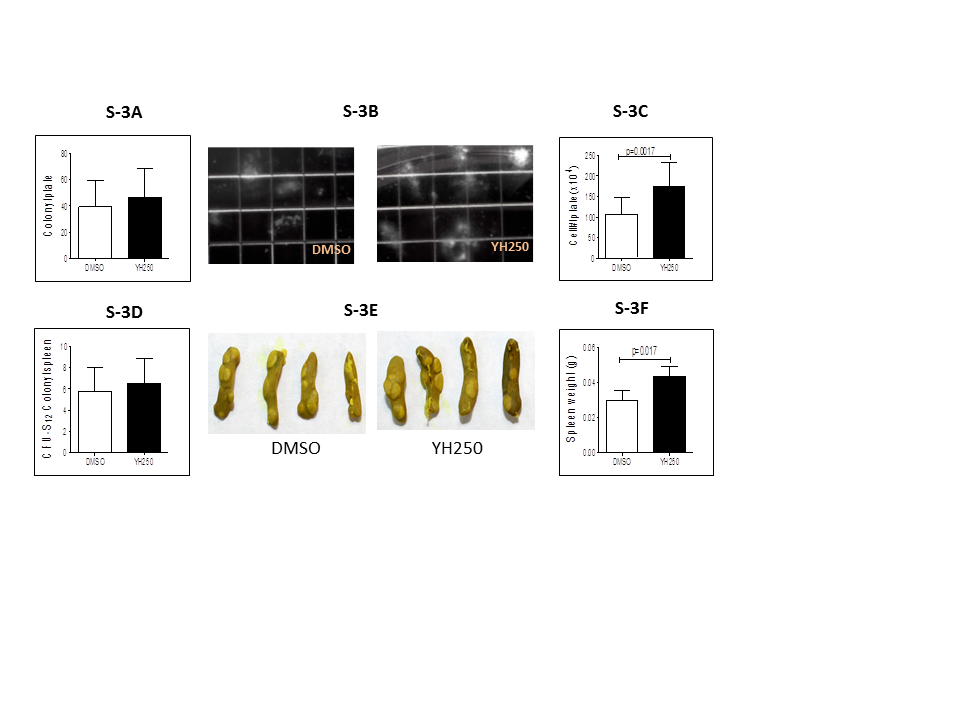

Supplement: S3 Fig — (A-C) CFC or (D-F) CFU-S12 assay with bone marrow cells treated with either DMSO or YH250 for 4 hours in vitro. Results shown are represents from 3 independent experiments. (TIF) [file pone.0177245.s003.TIF]
