# Supplementary material for: Small molecule p300/catenin antagonist enhances hematopoietic recovery after radiation
Source: PLoS One. 2017 May 9;12(5):e0177245. doi: 10.1371/journal.pone.0177245 (PMC5423697; doi:10.1371/journal.pone.0177245)
Supplement: S1 Table — (DOC) [file pone.0177245.s004.doc]

**S1 Table. RNA-seq analysis of gene differential expression in LSK150^+^48^-^ 41^-^** cells from YH250 vs DMSO treated animals

| **Chromosome** | **strand** | **gene_type** | **gene_symbol** | **Fold-Change** |
| --- | --- | --- | --- | --- |
| 4 | - | protein | Cd52 | 3.09 |
| 7 | + | protein | Cd79a | 5.49 |
| 11 | - | protein | Tmed4 | 2.06 |
| 12 | - | protein | Id2 | 3.97 |
| 15 | - | protein | Ly6c2 | 7.17 |
| 17 | + | protein | Ndufaf7 | 3.45 |
| 18 | + | protein | Cd74 | 2.30 |
| 1 | + | protein | Mrpl30 | 2.07 |
| 3 | - | protein | Car1 | 46.29 |
| 3 | + | protein | Casp6 | 2.56 |
| 6 | - | protein | Cd69 | 2.44 |
| X | - | protein | Il2rg | 2.03 |
| 9 | + | protein | Ldlr | -3.57 |
| 9 | - | protein | Pias1 | 2.02 |
| 2 | - | protein | Psmf1 | 2.48 |
| X | + | protein | Mid1 | -3.98 |
| 2 | - | protein | Cdc123 | 2.13 |
| 10 | - | protein | Dtx3 | -2.12 |
| 3 | - | protein | Cd53 | 2.79 |
| 4 | + | protein | A430005L14Rik | 2.07 |
| 5 | - | protein | Igj | 3.36 |
| 9 | - | miRNA | Gm26377 | -58.02 |
| 6 | + | IG_C_gene | Igkc | 3.93 |
| 12 | - | IG_C_gene | Ighm | 6.14 |
| 16 | - | protein | Iglc2 | 4.58 |
| 11 | - | protein | Tgtp1 | 2.85 |
| 6 | + | IG_LV_gene | Igkv14-126 | 8.00 |
| 16 | + | protein | Gm21897 | -2.18 |
| 12 | - | IG_LV_gene | Ighv11-2 | 8.18 |
| 6 | - | IG_LV_gene | Igkv19-93 | 21.16 |
| 7 | - | antisense | Kcnq1ot1 | -2.37 |
| X | + | lincRNA | RP23-99K18.3 | -2.28 |
